# Rapid genome sequencing for critically ill infants: an inaugural pilot study from Turkey

**DOI:** 10.3389/fped.2024.1412880

**Published:** 2024-07-04

**Authors:** Bengisu Guner Yilmaz, Ozlem Akgun-Dogan, Ozkan Ozdemir, Bayram Yuksel, Ozden Hatirnaz Ng, Kaya Bilguvar, Beril Ay, Gulsah Sebnem Ozkose, Eylul Aydin, Ayca Yigit, Aybike Bulut, Fatma Nisa Esen, Serdar Beken, Selma Aktas, Atalay Demirel, Baran Cengiz Arcagok, Ebru Kazanci, İbrahim Bingol, Ozge Umur, Guntulu Sik, Ugur Isik, Melike Ersoy, Ayse Korkmaz, Agop Citak, Adil Mardinoglu, Ugur Ozbek, Yasemin Alanay

**Affiliations:** ^1^ Department of Pediatrics, School of Medicine, Acibadem Mehmet Ali Aydinlar University, Istanbul, Turkey; ^2^ Division of Pediatric Genetics, Department of Pediatrics, School of Medicine, Acibadem Mehmet Ali Aydinlar University, Istanbul, Turkey; ^3^ Acibadem Mehmet Ali Aydinlar University Rare Diseases and Orphan Drugs Application and Research Center (ACURARE), Acibadem Mehmet Ali Aydinlar University, Istanbul, Turkey; ^4^ Department of Transitional Medicine, Health Sciences Institute, Acibadem Mehmet Ali Aydinlar University, Istanbul, Turkey; ^5^ Department of Genome Studies, Health Sciences Institute, Acibadem Mehmet Ali Aydinlar University, Istanbul, Turkey; ^6^ Division of Medical Biology, Department of Basic Sciences, School of Medicine, Acibadem Mehmet Ali Aydinlar University, Istanbul, Turkey; ^7^ Genetic Diagnosis Center, SZA OMICS, Istanbul, Turkey; ^8^ Department of Medical Genetics, School of Medicine, Acibadem Mehmet Ali Aydinlar University, Istanbul, Turkey; ^9^ School of Medicine, Acibadem Mehmet Ali Aydinlar University, Istanbul, Turkey; ^10^ Acibadem Labgen Genetic Diagnosis Center, Istanbul, Turkey; ^11^ Division of Neonatology, Department of Pediatrics, School of Medicine, Acibadem Mehmet Ali Aydinlar University, Istanbul, Turkey; ^12^ Division of Intensive Care, Department of Pediatrics, School of Medicine, Acibadem Mehmet Ali Aydinlar University, Istanbul, Turkey; ^13^ Division of Neurology, Department of Pediatrics, School of Medicine, Acibadem Mehmet Ali Aydinlar University, Istanbul, Turkey; ^14^ Division of Pediatric Metabolism, Department of Pediatrics, University of Health Sciences, Bakırkoy Dr. Sadi Konuk Training and Research, Istanbul, Turkey; ^15^ Faculty of Dentistry, Oral & Craniofacial Sciences, Centre for Host-Microbiome Interactions, King's College London, London, United Kingdom; ^16^ Science for Life Laboratory, KTH-Royal Institute of Technology, Stockholm, Sweden

**Keywords:** rapid genome sequencing, critically ill patient, pediatric intensive care unit, neonatal intensive care unit, trio sequencing

## Abstract

**Introduction:**

Rare and ultra-rare genetic conditions significantly contribute to infant morbidity and mortality, often presenting with atypical features and genetic heterogeneity that complicate management. Rapid genome sequencing (RGS) offers a timely and cost-effective approach to diagnosis, aiding in early clinical management and reducing unnecessary interventions. This pilot study represents the inaugural use of next-generation sequencing (NGS) as a diagnostic instrument for critically ill neonatal and pediatric ICU patients in a Turkish hospital setting.

**Methods:**

Ten infants were enrolled based on predefined inclusion criteria, and trio RGS was performed. The mean age of the participants was 124 days, with congenital abnormalities being the most common indication for testing. Three patients had consanguineous parents. The mean turnaround time from enrollment to delivery of results was 169 h, with a diagnostic yield of 50%.

**Results:**

Three patients received a definitive molecular diagnosis, impacting their clinical management. Two patients benefited from the exclusion of Mendelian conditions, leading to alternative diagnoses.

**Discussion:**

This study demonstrates the feasibility and results of RGS in Turkish hospital settings, emphasizing the importance of timely genetic diagnosis in reducing the diagnostic odyssey for families and improving patient care. Further research is needed to evaluate the cost-effectiveness and applicability of RGS in the Turkish healthcare system for children with diseases of uncertain etiology.

## 1 Introduction

Rare and ultra-rare genetic conditions serve as risk factors and direct causes of admissions to the intensive care unit, significantly impacting infant morbidity and mortality (1). The presence of accompanying comorbidities, atypical presentations, and clinic and genetic heterogeneities, when compounded by infancy and hospitalization in the intensive care unit, poses challenges for accurate diagnosis. The rapid progression of diseases in critically ill infants necessitates early genetic assessment to avoid unnecessary and potentially invasive, painful, and costly examinations and treatments. Early diagnosis not only facilitates clinical management but also alleviates anxiety among healthcare workers and addresses concerns within the family, ultimately reducing morbidity and mortality rates (2, 3). Recent advances in next-generation sequencing (NGS) techniques have decreased costs and time, along with increased availability (4). Over the last decade, more than 40 studies that involved over 3,500 critically ill pediatric patients suspected of having a genetic condition have used NGS for rapid diagnosis. Across these studies, the diagnosis time varied from rapid to ultra-rapid, and various NGS methods were employed. The reported diagnostic rates varied from 19% to 87.5%, with the duration of diagnosis spanning from 0.3 to 23 days. The impact of molecular diagnosis on clinical management varied from 30% to 100% (5–31).

These revolutionary findings have compelled organizations and governments to incorporate rapid genomics into clinical practice. Although some have achieved success in this endeavor (24, 28, 32, 33), even within the scope of a coverage policy and refund (34), others are currently integrating this advanced approach. In Turkey, newborn and infant mortality rates remain sufficiently high to warrant continued efforts in combating them (newborn: 5.7/1,000 and infant: 9.1/1,000) (35). According to the Organization for Economic Cooperation and Development, which ranks infant mortality rates for 48 countries, Turkey ranks ninth in this regard (36). Although the statistical breakdown of these deaths to identify underlying genetic causes is not yet known, it is established that 25.4% of the reported infant mortality cases in Turkey involve consanguinity between the parents (35). Considering that consanguineous marriage in Turkey is approximately 15 times more common than the European average and 6 times more common than the global average, the incidence and impact of genetic diseases are expected to be considerably higher (37).

This pilot study represents the inaugural use of NGS as a diagnostic instrument for critically ill neonatal and pediatric ICU patients in a Turkish hospital setting. The principal objectives of this investigation encompass the establishment and evaluation of a comprehensive workflow that integrates rapid NGS. Following established patient selection criteria from the literature, this study also assesses the diagnostic efficacy and impact of the diagnosis on patient care and determines the optimal timeframe required for this sequential cascade of events within our country's context.

## 2 Materials and methods

### 2.1 Patient selection and sample collection

The study was approved by the Ethics Committee of Acibadem University (ATADEK: 2021-23/30). This study prospectively enrolled 10 acutely deteriorating and critically ill infants between October 2022 and July 2023.

**Figure 1:**
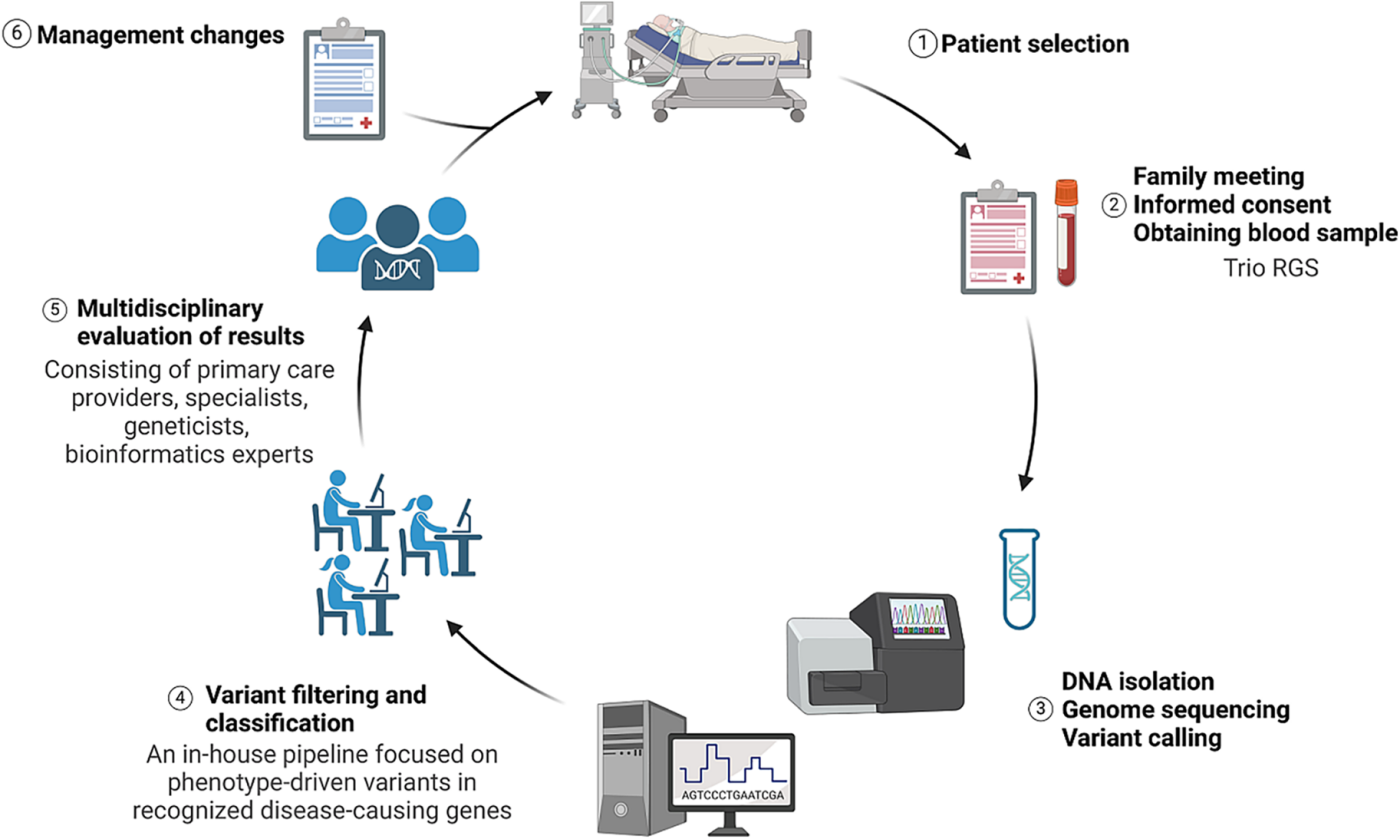
Illustration summarizing the study design from patient selection to management changes. Image conceived using Biorender.com.

Neonatologists and pediatric intensive care specialists from three Acibadem healthcare hospitals in Istanbul were involved in the study. They were tasked with referring acutely ill patients in need of a rapid diagnosis. Patient eligibility was assessed on a case-by-case basis by a multidisciplinary team (MDT) led by pediatric clinical geneticists. Other consulting specialists were invited according to the predefined inclusion criteria (Table 1). An immediate meeting was organized with the parents, the study was thoroughly explained, counseling was provided, and informed consent was obtained. Detailed family history and clinical data were obtained using a standard recording template. Furthermore, phenotypic information was listed as human phenotype ontology (HPO) terms. Blood samples (index–mother–father) were collected and transferred to the Acibadem University Biobank Study (Figure 1). Patients with recognizable genetic conditions, known trisomies, or other genetic diagnoses were excluded from this study. Genetic analysis was planned as our standard routine procedure.

**Table 1:** Inclusion and exclusion criteria for enrollment in the study.

Inclusion	Exclusion
Acutely deteriorating critically ill infant (<12 months) plus one or more of the following:	Current critical clinical status explained with a definitive diagnosis such as isolated prematurity, infection with normal response to treatment, trauma, and malignancy
Multi-system failure	
Unexplained neurological findings	
Unexplained metabolic abnormalities	Had a genetic diagnosis or clear indication of a specific syndrome
Malformations	
Insufficient/abnormal response to standard therapy for an underlying condition	Parents declined to participate

### 2.2 Whole genome sequencing

DNA isolation, quantification and quality check, library preparation, genome sequencing, and data analysis (alignment and variant calling) were performed by the SZAomics biotechnology and research laboratory. DNA was isolated using a QIAamp DNA Blood Mini Kit according to the manufacturer's instructions. Illumina DNA PCR-Free Prep Kit was used for the library preparation. Samples with sufficient QC scores were taken for sequencing. Whole genome sequencing (WGS) was performed on the Illumina NovaSeq 6000 (Illumina Inc., San Diego, CA, USA) system. Raw data (.blc) obtained after sequencing were converted to the Fast Adaptive Shrinkage Thresholding Algorithm and Quality (FASTQ) format via DRAGEN (v3.9.5). The binary alignment map file was obtained by aligning the FASTQ data to the GRCh38/hg38 human reference genome. The aligned raw data were converted to a VCF format from which the variants were called.

Data in a VCF format were annotated using GenNext developed in the laboratory of our university (https://github.com/GenivaInformatics/gennext-workflows/tree/main). Following variant annotation, filtering criteria were applied to obtain candidate variants. Variants were prioritized according to phenotype and pedigree information. *In silico* tools (CADD Exome, DANN Coding, FATHMM, MetaSVM, MutationTaster, PROVEAN, SIFT, MutationAssessor, MetaLR, PolyPhen-2, REVEL, and SpliceAI) were used to predict the pathogenicity of the variants. Population-, disease-, and sequence-specific open-source or paid databases were used in all variant analysis steps. The most frequently used databases were gnomAD3, 1000 Genomes Project, dbSNP, dbVar, ClinVar, OMIM, Human Gene Mutation Database, Human Genome Variation Society, DECIPHER, Leiden Open Variation Database, NCBI Genome, RefSeq, GME, Ensembl, Iranome, and Turkish Variome.

Variants with a reading depth of at least 95% for 30 times were evaluated. The identified variants were evaluated based on the variant identification guidelines provided by the Association for Clinical Genomic Science and Clinical Genome Resource and were further classified according to the American College of Medical Genetics and Genomics variant classification guide. Within this framework, variants categorized as “benign” and “possibly benign” were excluded from reporting. Only variants classified as “uncertain clinical significance (VUS)” pertaining to the patient's phenotype and all “likely pathogenic” and “pathogenic” variants were included in the report. The nomenclature of reported variants adheres to the Human Genome Variation Society guidelines.

### 2.3 MDT discussion and reporting

Following variant filtering and classification, a comprehensive meeting was convened, during which all team members discussed potential variants. All clinical consultants were present in the meeting. If a variant or variants contributing to the patient's phenotype were identified, a comprehensive report was generated. The results were communicated to the parents in person by the pediatric clinical geneticists. In cases where the result was negative, the family received counseling regarding the yearly reanalysis of the data.

## 3 Results

Ten critically ill infants were enrolled over a 9-month period. All patients underwent trio rapid genome sequencing (RGS). Siblings were included in two families. The mean age of the participants was 124 days, ranging from 2 days to 11 months. Six were female. An equal number of patients were referred from the neonatal intensive care unit (NICU) and pediatric intensive care unit (PICU). The most common indication for testing was congenital abnormalities. Three patients had consanguineous parents. Five patients had dysmorphic facial features.

**Figure 2:**
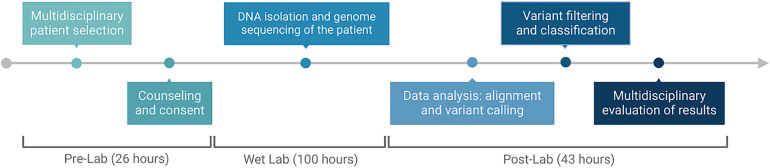
Timeline and process flow graph demonstrating each part of the workflow. Image conceived using Biorender.com.

The mean turnaround time (TAT) to the result (starting from sample receipt to sequencing report) was 169 h (range, 124–240 h). The average pre-lab time including multidisciplinary patient selection, counseling, and consent was 26 h, the wet-lab time including DNA isolation and genome sequencing was 100 h, and the post-lab time including data alignment, variant calling, variant filtering, and evaluation of results was 43 h (Figure 2). The final reports of cases with positive results were concluded faster: the average time was 147 h, whereas inconclusive or non-diagnostic results were reported at an average of 174.5 h.

**Figure 3:**
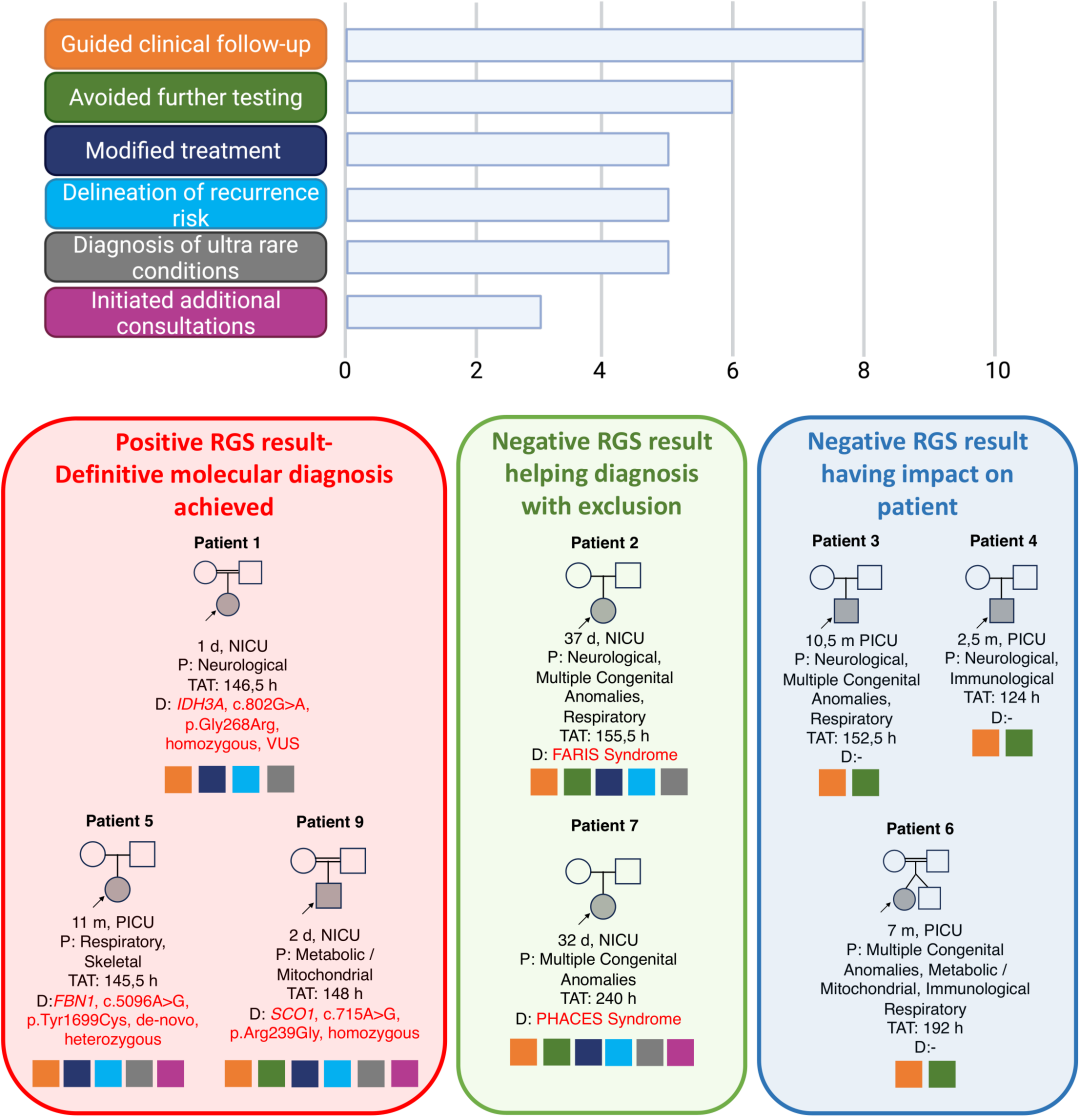
Overall implications of management in the 10 patients and their families. Image conceived using Biorender.com.

Table 2 summarizes the clinical indications, results, and TAT. Figure 3 shows the impact on management.

**Table 2:** Demographics, clinical summary, and RGS test results of the study cohort.

Patient	Ward	Gender	Age	Clinical features	Cons	Test	Gene	Final diagnosis	TAT (h)	Status
1	NICU	F	2 days	Seizure (HP:0001250), encephalopathy (HP:0001298), macular dystrophy (HP:0007754), abnormal cranial MRI (HP:0032615)	+	Trio	IDH3A	IDH3A-related mitochondrial encephalopathy	146.5	Alive
2	NICU	F	37 days	Hypotonia (HP:0001252), respiratory failure (HP:0002878), arthrogryposis multiplex congenita (HP:0002804), difficulty in sucking (HP:0002033), difficulty in feeding (HP:0011968)	–	Trio	–	FARIS (fetal acetylcholine receptor inactivation syndrome)	155.5	Alive
3	PICU	M	10.5 months	Multiple congenital anomalies (anal atresia (HP:0002023), choanal atresia (HP:0000453), hypoplastic aortic arch (HP:0012304), abnormal pulmonary venous return (HP:0010772), arrhythmia (HP:0011675), hydrocephalus (HP:0000238), ectopic kidney (HP:0000086)	–	Duo	–	Multiple congenital abnormalities—unidentified etiology	152.5	Alive
4	PICU	M	2.5 months	Seizure (HP:0001250), sepsis (HP:0100806), hemodynamic instability	–	Trio	–	Sepsis	124	Alive
5	PICU	F	11 months	Respiratory distress/failure (HP:0002098; HP:0002878), mitral stenosis (HP:0001718), subvalvular aortic stenosis (HP:0001682), acromicria (HP:0031878), abnormal midface morphology (HP:0000309), short stature (HP:0004322)	–	Trio	FBN1	Geleophysic dysplasia (#614185)	145.5	Deceased (post-test)
6	PICU	F	7 months	Transposition of great arteries (HP:0001669), lactic acidosis (HP:0003128), sepsis (HP:0100806), microcephaly (HP:0000252), abnormal thrombosis (HP:0001977), abnormal immunoglobulin levels (HP:0010701)	+	Trio	–	Isolated congenital heart defect	192	Deceased (post-test)
7	NICU	F	32 days	Coarctation of aorta (HP:0001680), microphthalmia (HP:0000568), bifid sternum (HP:0010309), supraumbilical raphe (HP:0410276), hemangioma (HP:0001028), optic nerve hypoplasia (HP:0000609)	–	Trio	–	PHACES (posterior fossa malformations, hemangioma, arterial anomalies, cardiac anomalies, eye anomalies, sternal defects)	240	Alive
8	PICU	F	7 months	Respiratory distress/failure (HP:0002098; HP:0002878), abnormal immunological findings (HP:0002715), Sepsis (HP:0100806), shock (HP:0031273), allergy (HP:0012393)	–	Trio	–	Septic shock	194	Deceased (post-test)
9	NICU	M	2 months	Low blood oxygen saturation (HP:0012418), metabolic acidosis (HP:0001942), lactic acidosis (HP:0003128), right ventricular hypertrophy (HP:0001667)	+	Trio	SCO1	Mitochondrial complex 4 deficiency, nuclear type 4 (#619048)	148	Alive
10	NICU	M	11 days	Prematurity, thrombocytopenia, coagulopathy, lactic acidosis (HP:0003128), hydronephrosis (HP:0000126), hemolytic anemia (HP:00001878), perirenal hematoma (HP:0030171)	–	Trio	–	Prematurity	193	Alive

NICU, neonatal intensive care unit; PICU, pediatric intensive care unit; Cons, consanguinity; TAT, turnaround time.

### 3.1 Definitive molecular diagnosis achieved in Patients 1, 5, and 9

#### 3.1.1 Patient 1

A 2-day-old female born at term as the second child of healthy first-cousin parents was admitted to the NICU due to suspicion of seizure. Electroencephalogram was abnormal. Cranial ultrasonography revealed a choroid plexus cyst and asymmetry of lateral ventricles. Trio RGS was performed on Day 2. The result was reported as negative in 146 h. The patient was discharged with antiepileptic medication and readmitted a month later due to intractable seizures. Eye examination revealed macular dystrophy. Cranial magnetic resonance imaging (MRI) revealed restricted diffusion in the bilateral perirolandic area and in the posterior crus of the internal capsule in the appropriate location to the corticospinal tract, hypomyelination, and widening of the subarachnoid distance in the bilateral frontal region. A re-evaluation of the RGS data with additional HPO terms, that is, “(HP:0007754) macular dystrophy,” led to the identification of a homozygous candidate variant in the *IDH3A* gene (ENST00000299518.7, c.802G>A, p.Gly268Arg) (OMIM:601149). The biallelic variants in the *IDH3A* gene are associated with retinitis pigmentosa 90 (#619007) in Online Mendelian Inheritance in Man (OMIM). However, a similar patient with retinitis pigmentosa, bilateral optic atrophy, and severe mitochondrial encephalopathy was previously reported (38). Therefore, the family was counseled regarding the diagnosis of this ultra-rare condition.

#### 3.1.2 Patient 5

An 11-month-old female born at term to unrelated healthy parents was previously evaluated by the pediatric genetics department due to short stature. The *FGFR3* gene analysis was negative, and the whole exome/genome sequencing analysis was planned. The patient was healthy and developing well until her admission to the PICU due to a lower respiratory tract infection, which rapidly evolved into respiratory insufficiency. Echocardiography revealed severe mitral and subaortic stenosis. Trio RGS revealed (TAT, 145 h) a heterozygous *de novo* pathogenic variant in the *FBN1* gene (ENST00000316623, c.5096A>G, p.Tyr1699Cys) (OMIM: 134797). The clinical findings were compatible with geleophysic dysplasia (#614185). The family was counseled regarding the diagnosis. The cardiovascular surgeons were also informed. They decided to postpone the valve replacement surgery because of the high postoperative morbidity and mortality rates in infancy. The family chose to have the cardiac surgery in another center where the patient died during the postoperative follow-up.

#### 3.1.3 Patient 9

A 2-day-old male born at term as the fifth child of healthy first-cousin parents was admitted to the NICU with metabolic acidosis, high lactate levels, and hypoxemia. Echocardiography revealed right ventricular hypertrophic cardiomyopathy and endomyocardial fibroelastosis. Rapid trio RGS identified a homozygous VUS variant in the *SCO1* gene (ENST00000255390.10, c.715A>G, p.Arg239Gly) (OMIM: 603644) (TAT, 148 h). The biallelic variants in the *SCO1* gene were associated with “mitochondrial complex 4 deficiency, nuclear type 4” (#619048), a metabolic disorder characterized by hypotonia, developmental delay, encephalopathy, hypertrophic cardiomyopathy, hepatomegaly, hepatic steatosis, hepatic failure, feeding difficulties, increased serum lactate, and metabolic acidosis. The family was counseled regarding this diagnosis; however, they were lost to follow-up after discharge.

### 3.2 Negative RGS result helping diagnosis with exclusion in Patients 2 and 7

#### 3.2.1 Patient 2

A 37-day-old female and the first child of unrelated parents was born at the 35th gestational week due to decreased fetal movements. The patient was admitted to the NICU due to hypotonia and poor respiratory effort. Transient neonatal myasthenia gravis (TNMG) was the initial diagnosis, supported by the mother's previous myasthenia gravis diagnosis. The patient was consulted for RGS because of her severe course unexpected for TNMG. The patient had a myopathic face, downward-sloping palpebral fissures, bulbous nose, severe feeding difficulty with no sucking or swallowing, and bilateral pes equinovarus. Trio RGS was performed, and the result was reported as negative in 155 h. The exclusion of other etiologies led to further consultation with international experts. Fetal acetylcholine receptor inactivation syndrome (FARIS) was suggested as a possible diagnosis (personal communication, Prof. Heinz Jungbluth). The analysis of fetal acetylcholine receptor antibodies (AchR) revealed high fetal AchR levels in the patient and the mother. The family was counseled regarding this diagnosis and the possibility of recurrence in future pregnancies.

#### 3.2.2 Patient 7

A 1-month-old male born at term as the first liveborn offspring of unrelated healthy parents was admitted to the NICU for prenatally diagnosed aortic coarctation. At birth, left microphthalmia, midline sternal defect, and paraumbilical raphe were detected. Eye examination revealed leukocoria and persistent hyperplastic vitreous in the left eye. Orbital MRI revealed retinal hemorrhage within the left vitreous, displaced optic lens, and left optic nerve atrophy. Trio RGS was performed, and the result was reported as negative in 240 h. The exclusion of other causes led to the diagnosis of PHACES syndrome (#606519). This prompted additional comprehensive radiological assessments, including cranial and thoracoabdominal MRI and MR angiography. The examination revealed the absence of the left internal carotid artery, with a retrograde flow detected in the left vertebral arteries. The family received counseling regarding this diagnosis, and treatment with acetylsalicylic acid was initiated.

### 3.3 Negative RGS result

Trio RGS was negative in Patients 3, 4, 6, 8, and 10.

## 4 Discussion

RGS, rapid exome sequencing, and rapid NGS panels are increasingly considered the first-line diagnostic tests for genetic diseases in ICU patients (4). Considering the increasing daily costs of care in NICUs and PICUs and sophisticated investigations during the diagnosis process, it is likely that the current cost savings per patient diagnosed by rapid sequencing are remarkable (39, 40). Retrospective and prospective studies using these methods in diverse cohorts have provided a diagnostic yield between 19% and 87.5% (5–31). This considerable variability is likely attributable to the differences in the inclusion criteria, particularly between studies using the same method.

This study is the first application of RGS in critically ill infants in Turkey. We successfully implemented an end-to-end protocol in 10 patients over 9 months. The trio RGS analysis results were achieved within 124–240 h (mean, 169 h). This TAT was comparable to that in previous studies conducted with RGS (7, 9, 21, 28). Infants with readily recognizable genetic conditions were excluded from this study. A genetic cause was identified in three patients, whereas a clinical diagnosis was established with the exclusion of the Mendelian conditions in two additional patients. We, therefore, suggest that the diagnostic yield in this study is 50%. All results were delivered back to the families and physicians involved in care before discharge from the hospital.

Technically, the trio WGS analysis, which surpasses single/trio whole exome sequencing (WES), offers numerous advantages, including enhanced copy number calling and the identification of mitochondrial and non-coding variants. Furthermore, it eliminates the time spent on the segregation analysis. However, note that WGS necessitates high-efficiency sequencers, along with expertise in bioinformatics and genetics within the field of translational genomics. Our study did not substantiate the superiority of WGS over WES. All causative variants identified in our study were coding SNVs, which would have been identified using a trio WES analysis. However, patients diagnosed with PHACES and FARIS benefited from a negative WGS result.

This study has several limitations. The small cohort size compared with previous studies in other countries was insufficient to provide conclusive data. The cost of trio RGS in a university-funded research project limited our ability to ensure a larger cohort. The data were analyzed using a phenotype-based prioritization approach, which might have restricted our analysis to a group of established genes, without a candidate gene approach. Finally, although this pilot study successfully prevented additional unnecessary tests for nearly all patients, note that the study design did not incorporate a formal cost-effectiveness analysis; therefore, the financial burden could not be estimated.

In conclusion, a prompt and timely diagnosis facilitated by rapid genetic testing can eliminate the diagnostic odyssey, enable targeted therapy, reduce the need for unnecessary invasive and costly interventions, alleviate uncertainty about the diagnosis for families, and facilitate family planning decisions. This inaugural study represents the first demonstration of the feasibility and results of RGS in critically ill infants in ICUs in Turkey. However, conducting additional research is crucial in evaluating the cost-effectiveness of such testing and in determining the widespread applicability for the entire Turkish national healthcare system. Future studies will determine the impact of the integration of rapid sequencing into the Turkish healthcare system for all children with diseases of uncertain etiology at ICU admission.

## Data Availability

The original contributions presented in the study are included in the article/Supplementary Material, further inquiries can be directed to the corresponding author.

## References

[1] Ferreira CR. The burden of rare diseases. Am J Med Genet A. (2019) 179(6):885–92. 10.1002/ajmg.a.61124 30883013

[2] Wu AC McMahon P Lu C. Ending the diagnostic odyssey—is whole-genome sequencing the answer? JAMA Pediatr. (2020) 174(9):821–2. 10.1001/jamapediatrics.2020.1522 PMC7928067 32597967

[3] Bauskis A Strange C Molster C Fisher C. The diagnostic odyssey: insights from parents of children living with an undiagnosed condition. Orphanet J Rare Dis. (2022) 17(1):233. 10.1186/s13023-022-02358-x 35717227 PMC9206122

[4] Kingsmore SF Nofsinger R Ellsworth K. Rapid genomic sequencing for genetic disease diagnosis and therapy in intensive care units: a review. NPJ Genom Med. (2024) 9(1):17. 10.1038/s41525-024-00404-0 38413639 PMC10899612

[5] Willig LK Petrikin JE Smith LD Saunders CJ Thiffault I Miller NA Whole-genome sequencing for identification of Mendelian disorders in critically ill infants: a retrospective analysis of diagnostic and clinical findings. Lancet Respir Med. (2015) 3(5):377–87. 10.1016/S2213-2600(15)00139-3 25937001 PMC4479194

[6] Clark MM Hildreth A Batalov S Ding Y Chowdhury S Watkins K Diagnosis of genetic diseases in seriously ill children by rapid whole-genome sequencing and automated phenotyping and interpretation. Sci Transl Med. (2019) 11(489). 10.1126/scitranslmed.aat6177 PMC9512059 31019026

[7] Wu B Kang W Wang Y Zhuang D Chen L Li L Application of full-spectrum rapid clinical genome sequencing improves diagnostic rate and clinical outcomes in critically ill infants in the China Neonatal Genomes Project. Crit Care Med. (2021) 49(10):1674–83. 10.1097/CCM.0000000000005052 33935161

[8] Elliott AM du Souich C Lehman A Guella I Evans DM Candido T RAPIDOMICS: rapid genome-wide sequencing in a neonatal intensive care unit-successes and challenges. Eur J Pediatr. (2019) 178(8):1207–18. 10.1007/s00431-019-03399-4 31172278

[9] Mestek-Boukhibar L Clement E Jones WD Drury S Ocaka L Gagunashvili A Rapid paediatric sequencing (RaPS): comprehensive real-life workflow for rapid diagnosis of critically ill children. J Med Genet. (2018) 55(11):721–8. 10.1136/jmedgenet-2018-105396 30049826 PMC6252361

[10] Brunelli L Jenkins SM Gudgeon JM Bleyl SB Miller CE Tvrdik T Targeted gene panel sequencing for the rapid diagnosis of acutely ill infants. Mol Genet Genomic Med. (2019) 7(7):e00796. 10.1002/mgg3.796 31192527 PMC6625092

[11] Daoud H Luco SM Li R Bareke E Beaulieu C Jarinova O Next-generation sequencing for diagnosis of rare diseases in the neonatal intensive care unit. CMAJ. (2016) 188(11):E254–60. 10.1503/cmaj.150823 27241786 PMC4978597

[12] Meng L Pammi M Saronwala A Magoulas P Ghazi AR Vetrini F Use of exome sequencing for infants in intensive care units: ascertainment of severe single-gene disorders and effect on medical management. JAMA Pediatr. (2017) 171(12):e173438. 10.1001/jamapediatrics.2017.3438 28973083 PMC6359927

[13] van Diemen CC Kerstjens-Frederikse WS Bergman KA de Koning TJ Sikkema-Raddatz B van der Velde JK Rapid targeted genomics in critically ill newborns. Pediatrics. (2017) 140(4):e20162854. 10.1542/peds.2016-2854 28939701

[14] Petrikin JE Cakici JA Clark MM Willig LK Sweeney NM Farrow EG The NSIGHT1-randomized controlled trial: rapid whole-genome sequencing for accelerated etiologic diagnosis in critically ill infants. NPJ Genom Med. (2018) 3:6. 10.1038/s41525-018-0045-8 29449963 PMC5807510

[15] Stark Z Lunke S Brett GR Tan NB Stapleton R Kumble S Meeting the challenges of implementing rapid genomic testing in acute pediatric care. Genet Med. (2018) 20(12):1554–63. 10.1038/gim.2018.37 29543227

[16] Farnaes L Hildreth A Sweeney NM Clark MM Chowdhury S Nahas S Rapid whole-genome sequencing decreases infant morbidity and cost of hospitalization. NPJ Genom Med. (2018) 3:10. 10.1038/s41525-018-0049-4 29644095 PMC5884823

[17] French CE Delon I Dolling H Sanchis-Juan A Shamardina O Megy K Whole genome sequencing reveals that genetic conditions are frequent in intensively ill children. Intensive Care Med. (2019) 45(5):627–36. 10.1007/s00134-019-05552-x 30847515 PMC6483967

[18] Kapil S Fishler KP Euteneuer JC Brunelli L. Many newborns in level IV NICUs are eligible for rapid DNA sequencing. Am J Med Genet A. (2019) 179(2):280–4. 10.1002/ajmg.a.61011 30569577

[19] Kernan KF Ghaloul-Gonzalez L Vockley J Carcillo JA. Rapid whole genome sequencing and fulfilling the promise of precision pediatric critical care. Pediatr Crit Care Med. (2019) 20(11):1085–6. 10.1097/PCC.0000000000002082 31688677 PMC7232857

[20] Kingsmore SF Cakici JA Clark MM Gaughran M Feddock M Batalov S A randomized, controlled trial of the analytic and diagnostic performance of singleton and trio, rapid genome and exome sequencing in ill infants. Am J Hum Genet. (2019) 105(4):719–33. 10.1016/j.ajhg.2019.08.009 31564432 PMC6817534

[21] Wang H Lu Y Dong X Lu G Cheng G Qian Y Optimized trio genome sequencing (OTGS) as a first-tier genetic test in critically ill infants: practice in China. Hum Genet. (2020) 139(4):473–82. 10.1007/s00439-019-02103-8 31965297

[22] Gubbels CS VanNoy GE Madden JA Copenheaver D Yang S Wojcik MH Prospective, phenotype-driven selection of critically ill neonates for rapid exome sequencing is associated with high diagnostic yield. Genet Med. (2020) 22(4):736–44. 10.1038/s41436-019-0708-6 31780822 PMC7127968

[23] Australian Genomics Health Alliance Acute Care Flagship, Lunke S Eggers S Wilson M Patel C Barnett CP Feasibility of ultra-rapid exome sequencing in critically ill infants and children with suspected monogenic conditions in the Australian public health care system. JAMA. (2020) 323(24):2503–11. 10.1001/jama.2020.7671 32573669 PMC7312414

[24] de Castro MJ Gonzalez-Vioque E Barbosa-Gouveia S Salguero E Rite S Lopez-Suarez O Rapid phenotype-driven gene sequencing with the NeoSeq panel: a diagnostic tool for critically ill newborns with suspected genetic disease. J Clin Med. (2020) 9(8):2362. 10.3390/jcm9082362 32718099 PMC7464859

[25] Smigiel R Biela M Szmyd K Bloch M Szmida E Skiba P Rapid whole-exome sequencing as a diagnostic tool in a neonatal/pediatric intensive care unit. J Clin Med. (2020) 9(7):2220. 10.3390/jcm9072220 32668698 PMC7408678

[26] Williamson SL Rasanayagam CN Glover KJ Baptista J Naik S Satodia P Rapid exome sequencing: revolutionises the management of acutely unwell neonates. Eur J Pediatr. (2021) 180(12):3587–91. 10.1007/s00431-021-04115-x 34143244 PMC8212268

[27] McDermott H Sherlaw-Sturrock C Baptista J Hartles-Spencer L Naik S. Rapid exome sequencing in critically ill children impacts acute and long-term management of patients and their families: a retrospective regional evaluation. Eur J Med Genet. (2022) 65(9):104571. 10.1016/j.ejmg.2022.104571 35842091

[28] Beaman M Fisher K McDonald M Tan QKG Jackson D Cocanougher BT Rapid whole genome sequencing in critically ill neonates enables precision medicine pipeline. J Pers Med. (2022) 12(11):1924. 10.3390/jpm12111924 36422100 PMC9694815

[29] Gorzynski JE Goenka SD Shafin K Jensen TD Fisk DG Grove ME Ultrarapid nanopore genome sequencing in a critical care setting. N Engl J Med. (2022) 386(7):700–2. 10.1056/NEJMc2112090 35020984

[30] Wells CF Boursier G Yauy K Ruiz-Pallares N Mechin D Ruault V Rapid exome sequencing in critically ill infants: implementation in routine care from French regional hospital’s perspective. Eur J Hum Genet. (2022) 30(9):1076–82. 10.1038/s41431-022-01133-7 35729264 PMC9436918

[31] Lumaka A Fasquelle C Debray FG Alkan S Jacquinet A Harvengt J Rapid whole genome sequencing diagnoses and guides treatment in critically ill children in Belgium in less than 40 hours. Int J Mol Sci. (2023) 24(4):4003. 10.3390/ijms24044003 36835410 PMC9967120

[32] NHS England. National Genomic Test Directory—Testing Criteria for Rare and Inherited Disease. 31. (2021). Available online at: https://www.england.nhs.uk/wp-content/uploads/2018/08/rare-and-inherited-disease-eligibility-criteria-v2.pdf (Accessed March 04, 2024).

[33] Michigan Department of Health and Human Services. Coverage of Rapid Whole Genome Sequencing (rWGS) Testing. (2021). Available online at: https://www.michigan.gov/mdhhs/-/media/Project/Websites/mdhhs/Folder4/Folder30/Folder3/Folder130/Folder2/Folder230/Folder1/Folder330/MSA_21-33.pdf?rev=a0926c6ef44d4c999d9061d443e52014&hash=2A5EFB5B77844A665B7C90FAD3E56576 (Accessed March 04, 2024).

[34] Blue Shield of California. Whole Exome and Whole Genome Sequencing for Diagnosis of Genetic Disorders. (2018). Available online at: https://www.blueshieldca.com/bsca/bsc/public/common/PortalComponents/provider/StreamDocumentServlet?fileName=PRV_WholeExome_Sequen.pdf (Accessed March 04, 2024).

[35] Turkish Ministry of Health. Sağlık Bakanlığının Kuruluşunun 100. Yılında Türkiye’de Bebek Ölümleri Durum Raporu. (2021). Available online at: https://hsgm.saglik.gov.tr/depo/birimler/cocuk-ergen-sagligi-db/Dokumanlar/Kitaplar/Saglik_Bakanliginin_Kurulusunun_100._Yilinda_Turkiyede_Bebek_Olumleri_Durum_Raporu.pdf (Accessed March 04, 2024).

[36] OECD. Infant Mortality Rates (Indicator). (2024). 10.1787/83dea506-en

[37] Temaj G Nuhii N Sayer JA. The impact of consanguinity on human health and disease with an emphasis on rare diseases. J Rare Dis. (2022) 1. 10.1007/s44162-022-00004-5

[38] Fattal-Valevski A Eliyahu H Fraenkel ND Elmaliach G Hausman-Kedem M Shaag A Homozygous mutation, p.Pro304His, in IDH3A, encoding isocitrate dehydrogenase subunit is associated with severe encephalopathy in infancy. Neurogenetics. (2017) 18(1):57–61. 10.1007/s10048-016-0507-z 28058510

[39] Sanford Kobayashi E Waldman B Engorn BM Perofsky K Allred E Briggs B Cost efficacy of rapid whole genome sequencing in the pediatric intensive care unit. Front Pediatr. (2021) 9:809536. 10.3389/fped.2021.809536 35141181 PMC8818891

[40] Goranitis I Wu Y Lunke S White SM Tan TY Yeung A Is faster better? An economic evaluation of rapid and ultra-rapid genomic testing in critically ill infants and children. Genet Med. (2022) 24(5):1037–44. 10.1016/j.gim.2022.01.013 35181209

